# Supplementary material for: Porous TiO2/Carbon Dot Nanoflowers with Enhanced Surface Areas for Improving Photocatalytic Activity
Source: Nanomaterials (Basel). 2022 Jul 23;12(15):2536. doi: 10.3390/nano12152536 (PMC9331435; doi:10.3390/nano12152536)
Supplement: Supplementary file 1 [file nanomaterials-12-02536-s001.zip › nanomaterials-1812267-supplementary.pdf]

nanomaterials

Porous TiO_2/Carbon Dot Nanoflowers with Enhanced Surface Areas for Improving Photocatalytic Activity

Fengyan Song [1], Hao Sun [2,3,4], Hailong Ma [2,3,4] and Hui Gao [2,3,4,*]

1 Center of Excellence for Environmental Safety and Biological Effects, Beijing Key Laboratory for Green Catalysis and Separation, Department of Chemistry and Biology, School of Life Science and Chemistry, Faculty of Environment and Life, Beijing University of Technology, Beijing 100124, China; fengyansong@bjut.edu.cn

2 Ningbo Institute of Technology, Beihang University, Ningbo 315100, China; hao.sun@buaa.edu.cn (H.S.); hailongma@buaa.edu.cn (H.M.)

3 School of Aeronautic Science and Engineering, Beihang University, Beijing 100191, China

4 Hangzhou Innovation Institute (Yuhang), Beihang University, Hangzhou 310052, China

* Correspondence: h.gao@buaa.edu.cn

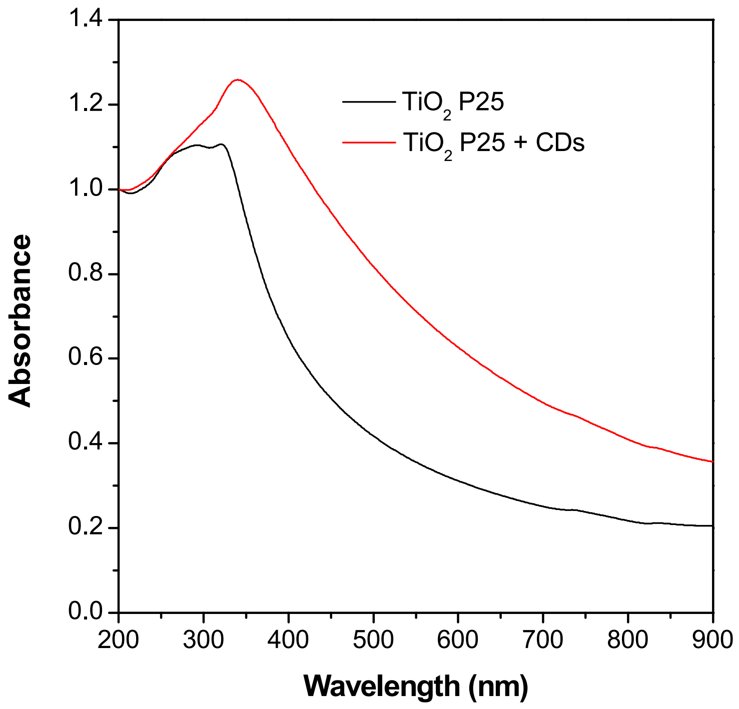

Figure S1. UV-Vis spectrums of pure P25 and P25 with CDs.

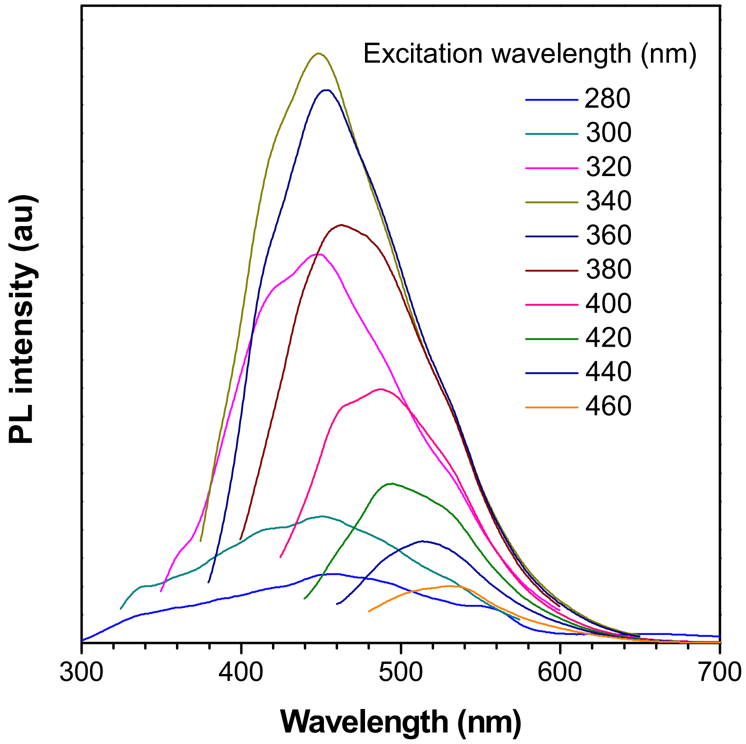

Figure S2. Photoluminescence spectrum of CDs with different excitation (add up-conversion).
